# The effect of ulinastatin on acute kidney injury in patients undergoing off-pump cardiac bypass surgery

**DOI:** 10.1186/s13019-024-02562-9

**Published:** 2024-02-15

**Authors:** Soo Jung Park, Sung Yong Park, Se Yoon Kang, Ji Ho Kim, Ji Yeong Heo, Ji Young Yoo

**Affiliations:** 1 Department of Anesthesiology and Pain Medicine, Ajou University School of Medicine, 164 Worldcup-Ro, Yeongtong-Gu, Suwon, Korea; 2 Department of Medical Sciences, Ajou University School of Medicine, Suwon, Korea

**Keywords:** Ulinastatin, Acute Kidney Injury, Creatinine, ICU stay, OPCAB, Urine output

## Abstract

**Background:**

Ulinastatin, an anti-inflammatory and antioxidant trypsin inhibitor, has shown potential in mitigating acute kidney injury (AKI) and reducing serum creatinine levels after various surgeries. This retrospective study aimed to evaluate the effects of ulinastatin on AKI in patients undergoing off-pump coronary artery bypass (OPCAB) surgery.

**Methods:**

We hypothesized that the administration of ulinastatin could prevent AKI in OPCAB. Electrical medical records were reviewed to identify OPCAB patients between January 2015 and June 2020. The utilization of ulinastatin was randomly determined and applied during this period. Acute kidney injury was defined according to the KDIGO guideline, and its incidence was compared between the ulinastatin administration group and the control group. To investigate the effect of ulinastatin on renal function, multivariate logistic regression analysis was used to calculate propensity scores for each group.

**Results:**

A total 454 OPCAB were performed, and after following inclusion and exclusion process, 100 patients were identified in the ulinastatin group and 303 patients in the control group. Using 1:2 propensity score matching, we analyzed 100 and 200 patients in the ulinastatin and control groups. The incidence of AKI was similar between the groups (2.5% for the control group, 2.0% for the ulinastatin group, p > 0.999). However, the serum creatinine value on the first post-operative day were significantly lower in the ulinastatin group compared to the control group (0.774 ± 0.179 mg/dL vs 0.823 ± 0.216 mg/dL, *P* = 0.040), while no significant differences were observed for the other time points (*P* > 0.05). The length of ICU stay day was significantly shorter in the ulinastatin group (2.91 ± 2.81 day vs 5.22 ± 7.45 day, respectively, *P* < 0.001).

**Conclusions:**

Ulinastatin did not have a significant effect on the incidence of AKI; it demonstrated the ability to reduce post-operative serum creatine levels at first post-operative day and shorten the length of ICU stay.

## Introduction

Acute kidney injury (AKI) is a common complication of cardiac surgery that has a substantial impact on patient prognosis [1]. Its incidence has been reported to range from 5 to 40% [2, 3]. In cases where cardiac surgery-associated AKI (CSA-AKI) occurs, 1–5% of patients require renal replacement therapy [4, 5], leading to an increased mortality rate of 1.4–30% [6–8]. The development of CSA-AKI is associated with various risk factors including hemodynamic instability, inflammatory conditions, metabolic disturbances, and exposure to nephrotoxic agents [7, 9]. Cardiopulmonary bypass (CPB) is an important risk factor for cardiac surgery [10]. Because CPB itself is an independent risk factor for AKI, the incidence of AKI is significantly lower with off-pump coronary artery bypass (OPCAB) [8, 11]. However, even without a pump, several factors such as advanced age, hypertension, hypoperfusion, and inotrope exposure contribute to AKI in OPCAB [11]. Consequently, the implementation of kidney protection strategies is crucial for OPCAB management**.** Despite numerous clinical trials, no pharmacological renal-protection strategy has been demonstrated to reduce the incidence of CSA-AKI.

Ulinastatin, a glycoprotein derived from human urine, possesses anti-inflammatory and anti-oxidant properties [12, 13]. It is widely used in patients with pancreatitis, sepsis, and multiorgan dysfunction [14, 15]. Previous studies demonstrated that ulinastatin reduces the incidence of AKI and serum creatinine elevation after heart, kidney and liver surgeries [16–18]**.** However, research exploring the potential effect of ulinastatin on AKI occurrence in patients with preserved renal function undergoing OPCAB is lacking. Therefore, the aim of this retrospective study was to investigate whether ulinastatin effectively prevents AKI in patients undergoing OPCAB.

## Methods

This study was approved by our institution’s institutional review board (AJRB-MED-MDB-20-057) and registered as a National Institutes of Health (NIH) clinical trial (NCT04473144). This study was conducted in accordance with the Declaration of Helsinki-2013. Because the study was retrospective in nature, the need for individual patient consent was waived. The results are reported in accordance with the Strengthening the Reporting of Observational Studies in Epidemiology (STROBE) statement.

### Data curation and study subject

We explored an electronic medical record system to identify patients who underwent elective OPCAB at Ajou University Hospital between January 2015 and June 2020. Clinical data were automatically extracted from the SMART Electronic Medical Record, a single-center registry with de-identified data from Ajou University Hospital. The hospital information system was used to collect pre-operative, intraoperative, and post-operative data, including patients' medical history and laboratory test results.

The exclusion criteria were as follows: patients under 19 years of age, patients with chronic renal failure or end-stage renal disease requiring renal placement therapy, urgent or emergency surgery, other combined surgeries, combined heart surgery that required CPB, preoperative use of inotropics, preoperative use of extracorporeal membrane oxygenation, or an intra-aortic balloon pump. The inclusion criteria were defined as adult patients over 19 and above who underwent elective OPCAB surgery between January 2015 and June 2020, and do not meet the exclusion criteria mentioned above. As this was a retrospective study, a prior sample size calculation was not performed.

### Patient allocation

From January 2015 to June 2020, ulinastatin was administered randomly without specific indications during OPCAB surgery at our institution. Upon retrospectively analyzing this practice, it was found that one-fourth of the patients received ulinastatin. Therefore, we selected patients falling under the indications for this study among those who received ulinastatin or not in this period and analyzed them accordingly.

### Anesthetic management

All patients underwent anesthesia induction, maintenance, and postoperative recovery management according to the standard protocol for cardiac surgery at our institution. Before induction, 5-lead ECG, radial arterial line, saturation monitoring, bispectral index (BIS), and infrared regional cerebral O_2_ saturation (INVOS) were applied to all patients. The induction of anesthesia was achieved using midazolam, sufentanil, and rocuronium, followed by intubation. After induction, a MAC catheter was inserted through the right internal jugular vein (IJV) to monitor parameters like pulmonary artery pressure (PAP), central venous pressure (CVP), cardiac output, and mixed venous oxygen saturation using a Swan-Gantz catheter. Through the left IJV, a double central catheter was inserted, connecting an 18-guage line for continuous drug infusion and a 16-guage line for bolus drug administration. A transesophageal echocardiography (TEE) probe was inserted to examine cardiac function. Anesthesia maintenance was achieved using sevoflurane and sufentanil, aiming to maintain the patient’s BIS in the range of 40 to 65. Only the ulinastatin group received an infusion of 300,000 units ulinastatin with 100 mL of normal saline for 15 min after induction.

The surgery was conducted as OPCAB surgery without the use of CPB. The left internal mammary artery was connected to the left descending artery, while other coronary arteries were connected to the ascending aorta using saphenous vein graft. Octopus stabilizer was used for surgical site stabilization.

The intraoperative hemodynamic goals were maintained with two different parts. During the process of coronary artery anastomosis, mean arterial pressure (MAP) was maintained above 60 mmHg. At other times, we targeted systolic blood pressure (SBP) in the range of 100 mmHg to 140 mmHg. The hypotension was defined as a MAP below 60 mmHg.

Norepinephrine was infused as a first line inotropic agent and fluid administration was adjusted based on cardiac output and TEE monitoring. Additional drugs such as dobutamine, vasopressin, and milrinone were administered as needed. All other procedures were performed in the same manner in both the ulinastatin and control groups.

After operation, the amounts of crystalloid, colloid, albumin administered were measured at 6 h, 24 h, and 48 h and evaluated cumulatively. Urine output was also evaluated in a similar manner as the amount of fluid intake. The occurrence of postoperative complications such as lung, wound, and arrhythmia were assessed in patients until discharge.

### Measured variables and study outcomes

Age, sex, and histories of hypertension, diabetes mellitus, chronic obstructive pulmonary disease, and coronary artery disease were recorded for each participant using an electronic medical record system. Several variables related to the pre-operative status were also investigated, such as ejection fraction (%), hematocrit (%), New York Heart Association (NYHA) functional class, and serum creatinine levels (mg/dL). The baseline creatinine level was defined as the most recent measurement taken within 7 days prior to surgery.

The amount of intravenous fluid (mL), red blood cell (RBC) transfusion (units), total blood loss (mL), and urine output (mL) were recorded during OPCAB. We also documented the incidence of intraoperative hypotension, and the administration and dosage of furosemide and inotropes during surgery. Hypotension was defined as systolic blood pressure (SBP) < 80 mmHg or mean arterial blood pressure (MBP) < 60 mmHg. As for post-operative parameters, serial assessments of serum creatinine levels were performed at the immediate post-operative and on the first day, second day, third day, and one week after surgery. Acute kidney injury was defined by KIDIGO guideline [19] as any of the following: increase in serum creatinine by 0.3 mg/dl or more within 48 h after operation, increase in serum creatinine to 1.5 times of baseline or more, which is known ore presumed to have occurred within the prior 7 days, urine volume are less than 0.5 ml/kg/h for 6 h. The lung complications included pneumonia, atelectasis, pleural effusion, and pulmonary edema. The length of hospital stay was calculated from admission to discharge, and the length of intensive care unit (ICU) stay was calculated from the day of operation to the day of transfer to the general ward.

The primary endpoint was the incidence of AKI that involved elevated serum creatinine or decreased urine output in accordance with the KDIGO guideline. As secondary endpoints, we analyzed the incidence of intraoperative hypotension, red blood cell (RBC) transfusion, intraoperative urine output, lung complications, length of ICU stay, and hospital stay during the follow-up period.

### Statistical analysis

All statistical analyses were performed using the R software (version 3.5.3; R Foundation for Statistical Computing, Vienna, Austria). As this was a retrospective study, a prior sample size calculation was not performed. Statistical significance was set at p < 0.05. 3. Continuous variables are presented as mean ± standard deviation and were compared using Student’s t-test because the sample size of each group was large enough to meet the central limit theorem. Categorical variables are presented as numbers (percentages) and were analyzed using the chi-square test or Fisher’s exact test according to the expected frequency of each contingency table.

To investigate the effect of ulinastatin administration on renal function, multivariate logistic regression analysis was used to calculate the propensity scores for each study group. Propensity score matching was performed in a 1:2 ratio between the ulinastatin and control groups, based on the calculated scores. Univariate and multivariate linear regression analyses were conducted to examine the factors associated with ICU stay duration. Bidirectional variable selection based on the Akaike information criterion (AIC) was employed to analyze the linear coefficients of the variables. For each final model, additional fitting was conducted with variables showing multicollinearity and their interactions.

## Results

Between January 2015 and June 2020, 454 OPCAB were performed at our institution.

**Fig. 1:**
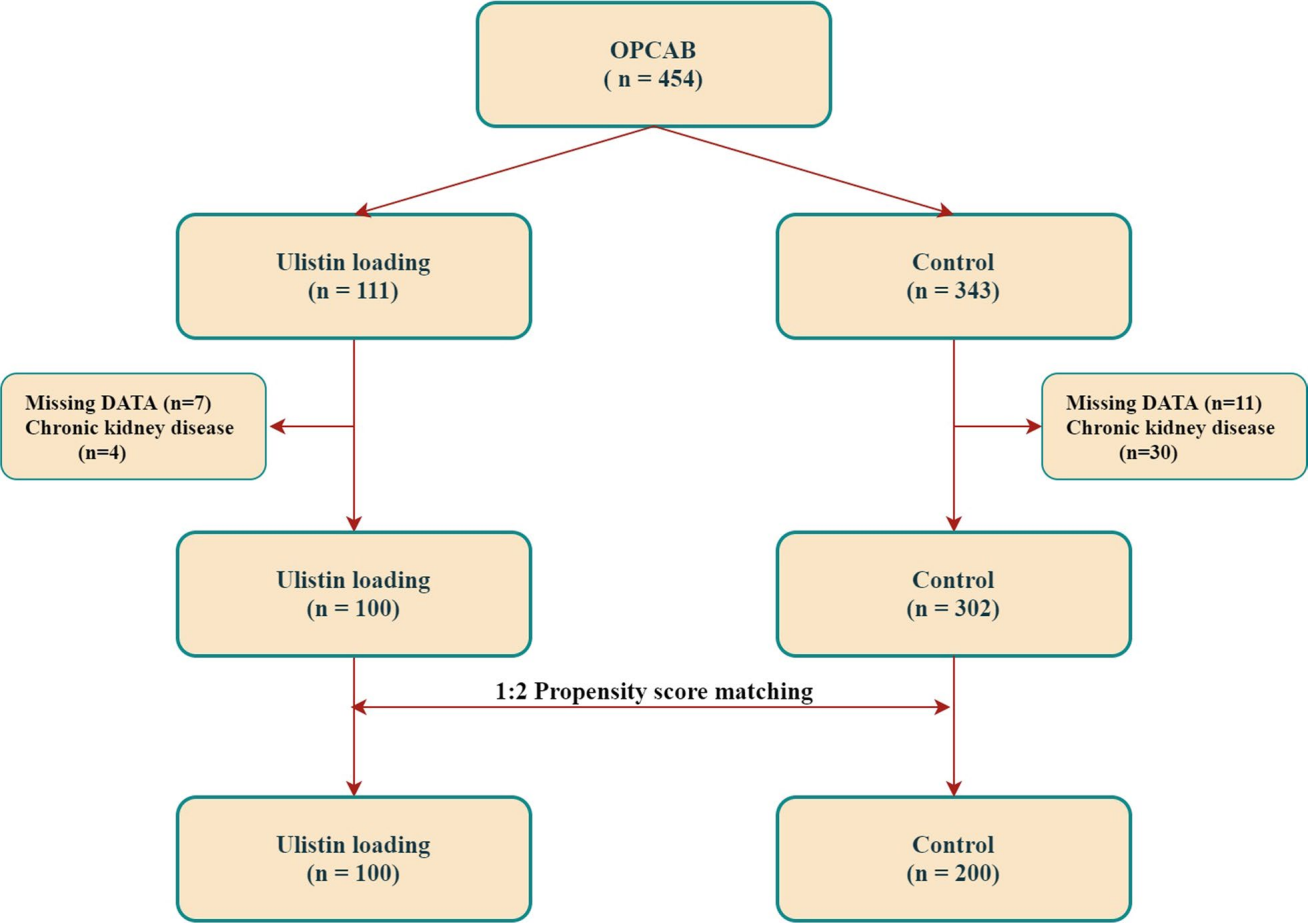
Patient flowchart

We identified 111 patients who underwent OPCAB following intra-operative ulinastatin administration (ulinastatin group). Among these patients, seven were excluded owing to missing data, and four were excluded for previously diagnosed chronic kidney disease. Using the same criteria, we identified 343 patients who underwent OPCAB without ulinastatin administration and assigned them to the control group**.** In the control group, 11 and 30 patients were excluded because of missing data and a history of chronic kidney disease, respectively. Throughout the inclusion and exclusion processes, 100 and 302 patients in the ulinastatin and control groups, respectively, were eligible for this investigation. Finally, 100 patients in the ulinastatin group and 200 in the control group were analyzed using a 1:2 coarsened exact matching process. A flowchart of the study is shown in Fig. 1.

The baseline characteristics and preoperative findings of patients treated with and without ulinastatin are presented in Table 1. The mean age, sex, and prevalence of hypertension, diabetes mellitus, and chronic obstructive pulmonary disease did not differ significantly between the two groups. The proportion of patients with an ejection fraction of less than 40% was equal in both groups (15%, p > 0.999). There was no statistical difference between the two groups in hematocrit (40 ± 5% for the control group, 40 ± 4% for the ulinastatin group, *p* = 0.987) or creatinine concentration (0.92 ± 0.21 mg/dL for the control group, 0.90 ± 0.20 mg/dL for the ulinastatin group; *p* = 0.638) (Table 1).

**Table 1:** Baseline characteristics and Preoperative Findings of Patients with and without Ulinastatin

	Control group (n = 200)	Ulinastatin group (n = 100)	*P* value
Age (years)	62.2 ± 11.5	61.8 ± 9.2	0.696
Sex (male)	171 (85.5%)	86 (86.0%)	> 0.999
Hypertension	127 (63.5%)	60 (60.0%)	0.643
Diabetes	85 (45.5%)	43 (43.0%)	> 0.999
COPD	2 (1.0%)	1 (1.0%)	> 0.999
EF	56.5 ± 14.2	56.4 ± 13.9	0.981
EF < 40%	30 (15.0%)	15 (15.0%)	> 0.999
Coronary a	2.8 ± 0.6	2.8 ± 0.5	0.601
LM coronary a	74 (37.0%)	41 (41.0%)	0.665
Hematocrit (%)	40.4 ± 5.0	40.4 ± 4.4	0.987
Creatinine (mg/dL)	0.915 ± 0.214	0.903 ± 0.200	0.638

Data are presented as n (%), mean ± standard deviation. *P* value < 0.05 indicates statistical significance

*COPD* Chronic obstructive pulmonary disease, *EF* Ejection fraction, *Coronary a.* Coronary artery with disease, *LM* Left main coronary artery with disease

The incidence of acute kidney injury was similar between the two groups (2.5% for the control group, 2.0% for the ulinastatin group, p > 0.99). However, there was a significant difference in the amount of intraoperative urine between the two groups (550.0 mL [385.0, 990.0] for the control group, 905.0 mL [615.0, 1235.0] for the ulinastatin group, *p* = 0.002) (Table 2). At post-operative 6 h, 24 h, and 48 h, there was no significant difference in the amount of urine between the two groups (*P* = 0.079, *P* = 0.371 and *P* = 0.516 respectively). Notably, there was a substantial difference in fluid administration between the two groups. During intraoperative period, crystalloid administration was significantly higher in the ulinastatin group than the control group (2650.0 mL [2100.0, 3250.0] vs 3100.0 mL [2540.0, 3900.0], *P* < 0.001). This corresponded to the difference in intraoperative urine output. There were no differences in the amount of colloid or albumin at intraoperative period (*P* = 0.771 and *P* = 0.356, respectively).

**Table 2:** Intraoperative and postoperative findings of patients with and without ulinastatin

	Control group (n = 200)	Ulinastatin group (n = 100)	*P* value
Blood loss (mL)	429.0 (300.0, 800.0)	450.0 (300.0, 500.0)	*0.375*
Fluid administration (mL)			
Intraoperative period			
Crystalloid	2650.0 (2100.0, 3250.0)	3100.0 (2540.0, 3900.0)	< 0.001*
Colloid	500.0 (0.0, 700.0)	500.0 (400.0, 550.0)	*0.771*
Albumin	0.0 (0.0, 200.0)	0.0 (0.0, 100.0)	*0.356*
Post-operative within 6 h			
Crystalloid	1100.0 (945.0, 1355.0)	1092.0 (890.0, 1292.0)	*0.348*
Colloid	0.0 (0.0, 0.0)	0.0 (0.0, 0.0)	*0.764*
Albumin	100.0 (88.0, 100.0)	93.0 (70.0, 100.0)	0.014*
Post-operative within 24 h			
Crystalloid	3835.0 (3373.0, 4230.0)	3605 (3235.0, 4126.0)	*0.055*
Colloid	0.0 (0.0, 0.0)	0.0 (0.0, 0.0)	*0.829*
Albumin	197.0 (95.0, 342.0)	100.0 (91.0, 258.0)	0.040*
Post-operative within 48 h			
Crystalloid	5945.0 (5552.0, 6810.0)	5880.0 (5111.0, 6390.0)	*0.053*
Colloid	0.0 (0.0, 0.0)	0.0 (0.0, 0.0)	*0.841*
Albumin	250.0 (100.0, 414.0)	235.0 (100.0, 346.0)	*0.260*
Number of intraoperative hypotension event	1.45 ± 1.66	1.50 ± 1.71	*0.807*
RBC transfusion (unit)	0.860 ± 1.228	0.850 ± 1.140	*0.946*
Intraoperative urine (mL)	550.0 (385.0, 990.0)	905.0 (615.0, 1235.0)	0.002*
Post-operative urine within 6 h	860.0 (575.0, 1275.0)	975.0 (760.0, 1330.0)	*0.079*
Post-operative urine within 24 h	2315.0 (1880.0, 3400.0)	2565.0 (2015.0, 3270.0)	*0.371*
Post-operative urine within 48 h	4460.0 (3585.0, 5510.0)	4285.0 (3710.0, 5380.0)	*0.516*

Data are presented as number, mean ± standard deviation, and median (interquartile range)

*P* value* < 0.05 indicates statistical significance

Intraoperative hypotension is defined as SBP ≤ 80 mmHg or MAP ≤ 60 mmHg

*RBC* Red blood cell

At post-operative 6 h and 24 h, there was no significant difference in the amount of crystalloid or colloid between the two groups. However, there was a notable difference in the administration of albumin, with more administered in the control group than in the ulinastatin group (100.0 mL [88.0, 100.0] for the control group, 93.0 mL [70.0, 100.0] for the ulinastatin group at post-operative 6 h, *P* = 0.014). At post-operative 24 h, the administration of albumin is statistically different (197.0 mL [95.0, 342.0] for the control group, 100.0 mL [91.0, 258.0] for the ulinastatin group, *P* = 0.040). However, at post-operative 48 h, there were no significant differences the amount of crystalloid, colloid, and albumin between the two groups. The amount of blood loss, and RBC transfusion did not significantly differ between the control and ulinastatin groups (*P* = 0.375, and 0.946, respectively). Intraoperative hypotension occurred at a similar rate in both the groups (*P* = 0.807) (Table 2).

The usage of norepinephrine, vasopressin, dobutamine, and milrinone during operation, immediate post operation, post-operative day1, post-operative day2 and post-operative day3 are presented in Table 3. Notably, there was no statistically significant difference between the two groups in terms of the number of patients receiving inotropic agents (Table 3).

**Table 3:** Intraoperative and post-operative inotropic agent usages

	Control group (n = 200)	Ulinastatin group (n = 100)	*P* value
*Intraoperative usage*			
Norepinephrine	154 (77.0%)	78 (78.0%)	> *0.999*
Vasopressin	14 (7.0%)	6 (6.0%)	*0.774*
Dobutamine	4 (2.0%)	1 (1.0%)	> *0.999*
Milrinone	8 (4.0%)	6 (6.0%)	*0.517*
*Immediate post operation*			
Norepinephrine	60 (30.0%)	28 (28.0%)	*0.706*
Vasopressin	0 (0.0%)	0 (0.0%)	*1.000*
Dobutamine	0 (0.0%)	1 (1.0%)	*1.000*
Milrinone	14 (7.0%)	4 (4.0%)	*0.234*
*Post-operative day 1*			
Norepinephrine	122 (61.0%)	53 (53.0%)	*0.263*
Vasopressin	4 (2.0%)	0 (0.0%)	*0.121*
Dobutamine	2 (1.0%)	2 (2.0%)	*1.000*
Milrinone	24 (12.0%)	8 (8.0%)	*0.347*
*Post-operative day 2*			
Norepinephrine	76 (38.0%)	33 (33.0%)	*0.468*
Vasopressin	4 (2.0%)	1 (1.0%)	*0.369*
Dobutamine	0 (0.0%)	1 (1.0%)	*1.000*
Milrinone	14 (7.0%)	5 (5.0%)	*0.552*
*Post-operative day 3*			
Norepinephrine	50 (25.0%)	20 (20.0%)	*0.407*
Vasopressin	1 (0.5%)	0 (0.0%)	*1.000*
Dobutamine	2 (1.0%)	2 (2.0%)	*1.000*
Milrinone	12 (6.0%)	2 (2.0%)	*0.279*

Data are presented as n (%)

*P* value < 0.05 indicates statistical significance

Serum creatinine levels were serially measured in all patients at baseline (Cr0) as well as on post-operative days 1 (Cr1), day2 (Cr2), day3 (Cr3), and day7 (Cr7). Among these values, Cr1 was significantly lower in the ulinastatin group (0.77 ± 0.18) compared to the control group (0.82 ± 0.22, *P* = 0.040), while others did not show statistical significance. The length of ICU stay was significantly shorter in the ulinastatin group (2.9 ± 2.8) than the control group (5.2 ± 7.5, *P* < 0.001). Although the mean length of total hospital day was slightly shorter in the ulinastatin group (15 ± 8) than the control group (16 ± 9), it did not reach statistical significance (*P* = 0.222) After operation period, there was no significant difference in lung complications, the incidence arrhythmia, and wound complications (Table 4).

**Table 4:** Post-operative outcome in patients with and without ulinastatin

Postoperative outcome	Control group (n = 200)	Ulinastatin group (n = 100)	*P* value
Acute kidney injury	5 (2.5%)	2 (2.0%)	> 0.999
*Creatine level(mg/dL)*			
Cr0	0.734 ± 0.176	0.734 ± 0.172	0.985
Cr1	0.823 ± 0.216	0.774 ± 0.179	0.040*
Cr2	0.766 ± 0.239	0.748 ± 0.186	0.453
Cr3	0.779 ± 0.304	0.753 ± 0.198	0.374
Cr7	0.832 ± 0.292	0.821 ± 0.193	0.687
Lung complication (%)	30 (15.0%)	12 (12.0%)	0.596
Postop arrhythmia (%)	16 (8.0%)	7 (7.0%)	0.788
Wound complications (%)	19 (9.5%)	8 (8.0%)	0.621
Length of hospital day(day)	15.8 ± 8.5	14.6 ± 7.1	0.222
Length of ICU stay(day)	5.22 ± 7.45	2.91 ± 2.81	< 0.001*

Data are presented as n (%) or mean ± standard deviation

*P* value* < 0.05 indicates statistical significance

*Cr0* Immediate post operation, *Cr1* post-operative day 1, *Cr2* post-operative day 2, *Cr3* post-operative day 3, *Cr7* post-operative day 7, *ICU* Intensive care unit

To elucidate the role of ulinastatin in Cr1, multivariate linear regression analysis was performed to assess potential interactions with other clinical variables (Table 5) Single-variable regression revealed that age, sex, lower EF (< 40%), initial serum creatinine level, number of intraoperative hypotension events, and intraoperative urine volume were significantly correlated with Cr1. However, multivariate linear regression analysis using the Bayesian information criterion for model fitting showed that ulinastatin administration was effective in reducing Cr1 by 0.041 (*P* = 0.017). Additionally, age, sex, lower EF (< 40%), initial hematocrit level, and initial creatinine level were independent factors that affected Cr1 levels. Regarding the length of ICU stay, single-variable linear regression analysis revealed significant correlations with ulinastatin use, lower EF < 40%, and initial creatinine level. However, in the multivariate linear regression analysis with the Bayesian information criterion for model fitting, only ulinastatin and a lower EF < 40% were identified as independent factors affecting the length of ICU stay. The administration of ulinastatin shortened the ICU stay by 2.3 days (*P* = 0.002), and patients with a lower EF (< 40%) spent an additional 4.3 days in the ICU (*P* < 0.001), regardless of other clinical conditions (Table 6).

**Table 5:** Univariate and multivariate regression analysis for factors affecting Cr1 in patients treated with ulinastatin

	Univariate		Multivariate	
	Coefficient ± SE	*P* value	Coefficient ± SE	*P* value
Ulinastatin	− 0.049 ± 0.025	0.053	− 0.041 ± 0.017	0.017*
Age (years)	0.0038 ± 0.0011	< 0.001*	0.0032 ± 0.0008	< 0.001*
Sex (Male)	− 0.22 ± 0.03	< 0.001*	− 0.14 ± 0.03	< 0.001*
Hypertension	0.042 ± 0.024	0.088	Eliminated	
Diabetes	0.047 ± 0.024	0.052	Eliminated	
COPD	0.095 ± 0.119	0.429	Eliminated	
EF < 40%	0.107 ± 0.033	0.001*	0.073 ± 0.023	0.001*
Hematocrit	− 0.00039 ± 0.00247	0.876	− 0.00473 ± 0.00182	0.010*
Baseline Creatinine (mg/dL)	0.67 ± 0.04	< 0.001*	0.56 ± 0.04	< 0.001*
Blood loss (mL)	− 0.029 ± 0.038	0.447	Eliminated	
Input fluid (mL)	0.00052 ± 0.01108	0.963	Eliminated	
Number of Intraoperative hypotension event	0.0086 ± 0.0071	0.002*	Eliminated	
RBC transfusion (unit)	0.011 ± 0.010	0.261	Eliminated	
Urine (mL)	− 0.060 ± 0.025	0.018*	Eliminated	

*P* value* < 0.05 indicates statistical significance

*Cr1* post-operative day 1, *COPD* Chronic obstructive pulmonary disease, *EF* Ejection fraction *RBC* Red blood cell

**Table 6:** Univariate and multivariate linear regression analysis for factors affecting length of ICU stay in patients treated with ulinastatin

	Univariate		Multivariate	
	Coefficient ± SE	*P* value	Coefficient ± SE	*P *value
Ulinastatin	− 2.31 ± 0.77	0.003*	− 2.31 ± 0.75	0.002*
Age (years)	0.062 ± 0.34	0.068	Eliminated	
Sex (Male)	− 0.23 ± 1.05	0.830	Eliminated	
Hypertension	0.49 ± 0.76	0.516	Eliminated	
Diabetes	1.38 ± 0.74	0.064	Eliminated	
COPD	− 0.79 ± 3.71	0.831	Eliminated	
EF < 40%	4.28 ± 1.00	< 0.001*	4.28 ± 0.99	< 0.001*
Hematocrit	− 0.099 ± 0.076	0.197	Eliminated	
Baseline creatinine(mg/dL)	4.11 ± 1.75	0.020*	Eliminated	
Blood loss (mL)	0.0010 ± 0.0011	0.377	Eliminated	
Input fluid (mL)	0.43 ± 0.34	0.209*	Eliminated	
Number of intraoperative hypotension event	0.41 ± 0.22	0.064	Eliminated	
RBC transfusion (unit)	0.44 ± 0.31	0.157	Eliminated	
Urine (mL)	0.77 ± 0.79	0.329	Eliminated	

*P* value* < 0.05 indicates statistical significance

*COPD* Chronic obstructive pulmonary disease, *EF* Ejection fraction *RBC* Red blood cell

## Discussion

In this study, we investigated the incidence of AKI in patients who underwent OPCAB and received ulinastatin. While a reduction in creatinine on post-operative day 1 was observed, propensity score matching revealed that ulinastatin did not significantly reduce the incidence of AKI. Furthermore, despite a statistically significant increase in intraoperative urine output was observed in the ulinastatin group, it was associated with notably higher administration of crystalloid. However, patients who received ulinastatin had a significantly shorter length of stay in the ICU, which was further supported by the multivariate logistic regression analysis. These findings provide information on the potential utility of ulinastatin in patients undergoing OPCAB.

AKI has been reported as a common complication of cardiac surgery and is associated with increased mortality and prolonged ICU stay [3, 9, 11]. The pathophysiology of CSA-AKI is multifactorial. Significant hemodynamic alterations, systemic inflammation, and metabolic changes occur during cardiac surgery, contributing to the development of CSA-AKI. Moreover, systemic inflammatory responses and oxidative stress are considered major pathophysiological mechanisms in the development of CSA-AKI, particularly after cardiac surgeries involving CPB [20, 21]. OPCAB surgery is a technique aimed at minimizing CPB-associated complications. However, even in OPCAB, a systemic inflammatory response and oxidative stress can occur due to factors such as hypoperfusion, blood loss, and endotoxemic ischemia–reperfusion injury [22–24]. Therefore, it is crucial to reduce systemic inflammatory responses and oxidative stress during OPCAB to prevent AKI.

Ulinastatin is a glycoprotein and protease inhibitor extracted from human urine [14]. The organ-protective effects of ulinastatin have been extensively explored in various animal studies. In rat models subjected to renal ischemia/reperfusion, ulinastatin administration was found to suppress the secretion of inflammatory cytokines such as TNF-a and IL-6, while also inhibiting the overproduction of reactive oxygen species (ROS), thereby reducing oxidative stress [12, 13, 25]. Similarly, in animal models of hepatic ischemia/reperfusion, ulinastatin administration was associated with the attenuation of inflammatory responses and the inhibition of neutrophil accumulation, as observed through pathological tissue examinations [26]. Based on the results of these animal experiments, several studies have been reported that investigate the effects of ulinastatin's multi-organ protection in various clinical scenarios, including patients with sepsis, acute lung injury, and other conditions. According to a propensity score-matched study conducted by Wan et al., ulinastatin administration significantly reduced the incidence of acute kidney injury (AKI) during surgeries utilizing CPB [16]. Furthermore, a study involving 174 patients undergoing liver transplantation concluded that those receiving high-dose ulinastatin had a lower frequency of late-onset acute renal failure [27]. A systematic review with a meta-analysis conducted in 2014, involving patients with ARDS and acute lung injury, demonstrated a shorter ICU stay in the ulinastatin-treated group [28]. Additionally, an RCT conducted with patients undergoing cardiac surgery also supported this observation by reporting a decrease in ICU length of stay associated with ulinastatin administration [29].

However, our data showed that although there was a decrease in 24-h creatinine levels, and a reduction in ICU length of stay following ulinastatin administration during surgery, there was no significant difference in the incidence of AKI. These findings are consistent with those of a study conducted by Lee et al., which reported that ulinastatin did not significantly reduce AKI in robot-assisted nephrectomy [17], as well as a study in patients undergoing aortic valve replacement, in which ulinastatin administration did not show a difference in serum creatinine levels [30]. One possible explanation for this phenomenon is the difference in the administered dose of ulinastatin. Previous studies reporting the clinical efficacy of ulinastatin involved single or multiple administrations of total 5000–10,000 U/kg or more than 1,000,000 units [16, 27, 31, 32]. In this study, to minimize potential side effects, such as leukopenia or hypersensitivity, a recommended dose of 300,000 IU was mixed with normal saline and administered immediately after induction [33]. While these dosage differences might have contributed to reducing the length of ICU stay and attenuating the inflammation in certain organs, they could have insufficient in preventing AKI. Additionally, in this study, ulinastatin was only administered immediately after induction, and if it had been administered in close proximity to the point where hypoperfusion and reperfusion injury were most severe or repeated, it could have affected renal function beyond 24 h post-surgery. Furthermore, because this study was conducted on OPCAB cases, which are associated with relatively less severe inflammatory responses and ischemic injuries, the effects of ulinastatin may not have been pronounced. Therefore, to evaluate the effectiveness of ulinastatin in OPCAB more accurately, further prospective studies with varying doses and administration times of ulinastatin are needed.

This study has the strength of comparing of AKI occurrence using KDIGO criteria; however, there were certain limitations to our study. First, as with all retrospective observational studies, there is a possibility of hidden bias and unmeasured confounding factors despite our efforts to address this through propensity score matching. Second, our study was conducted at a single center with a predominantly Asian patient population, which may limit the generalizability of our findings to other populations and settings. Third, we only analyzed the incidence of AKI and were unable to conduct additional analyses to evaluate the severity of AKI, such as the proportion of patients who received dialysis. Lastly, due to the retrospective design, we were unable to assess certain inflammatory laboratory markers, such as interleukin and TNF. Additionally, it was challenging to access parameters related to mechanical ventilator usage, changes in consciousness such as delirium, occurrences of infection, and the use of antibiotics, all of which are associated with ICU length of stay.

Our findings have investigated the potential utility of ulinastatin in preventing AKI in patients after OPCAB. Further prospective studies with larger and more diverse patient populations are needed to assess the effectiveness of preventive measures against AKI in patients undergoing OPCAB.

## Abbreviations

AKI: Acute kidney injury
AIC: Akaike information criterion
CSA-AKI: Cardiac surgery-associated AKI
CPB: Cardiopulmonary bypass
COPD: Chronic obstructive pulmonary disease
Cr: Creatinine
EF: Ejection fraction
ICU: Intensive care unit
OPCAB: Off-pump coronary artery bypass
MBP: Mean arterial blood pressure
RBC: Red blood cell
ROS: Reactive oxygen species
SBP: Systolic blood pressure

## References

[1] Bove T, Monaco F, Covello RD, Zangrillo A (2009). Acute renal failure and cardiac surgery. HSR Proc Intensive Care Cardiovasc Anesth.

[2] Bastin AJ, Ostermann M, Slack AJ, Diller GP, Finney SJ, Evans TW (2013). Acute kidney injury after cardiac surgery according to risk/injury/failure/loss/end-stage, acute kidney injury network, and kidney disease: improving global outcomes classifications. J Crit Care.

[3] Bove T, Calabro MG, Landoni G, Aletti G, Marino G, Crescenzi G (2004). The incidence and risk of acute renal failure after cardiac surgery. J Cardiothorac Vasc Anesth.

[4] Huen SC, Parikh CR (2012). Predicting acute kidney injury after cardiac surgery: a systematic review. Ann Thorac Surg.

[5] Karkouti K, Wijeysundera DN, Yau TM, Callum JL, Cheng DC, Crowther M (2009). Acute kidney injury after cardiac surgery: focus on modifiable risk factors. Circulation.

[6] Kuitunen A, Vento A, Suojaranta-Ylinen R, Pettila V (2006). Acute renal failure after cardiac surgery: evaluation of the RIFLE classification. Ann Thorac Surg.

[7] Marco PS, Nakazone MA, Maia LN, Machado MN (2022). Cardiac surgery-associated acute kidney injury in patients with preserved baseline renal function. Braz J Cardiovasc Surg.

[8] Hu J, Chen R, Liu S, Yu X, Zou J, Ding X (2016). Global incidence and outcomes of adult patients with acute kidney injury after cardiac surgery: a systematic review and meta-analysis. J Cardiothorac Vasc Anesth.

[9] Wang Y, Bellomo R (2017). Cardiac surgery-associated acute kidney injury: risk factors, pathophysiology and treatment. Nat Rev Nephrol.

[10] Lopez-Delgado JC, Esteve F, Torrado H, Rodriguez-Castro D, Carrio ML, Farrero E (2013). Influence of acute kidney injury on short- and long-term outcomes in patients undergoing cardiac surgery: risk factors and prognostic value of a modified RIFLE classification. Crit Care.

[11] Nigwekar SU, Kandula P, Hix JK, Thakar CV (2009). Off-pump coronary artery bypass surgery and acute kidney injury: a meta-analysis of randomized and observational studies. Am J Kidney Dis.

[12] Wang Y, Peng C, Zhang Z, Shi J, Lin Y, Gu L (2019). Intravenous infusion of ulinastatin attenuates acute kidney injury after cold ischemia/reperfusion. Int Urol Nephrol.

[13] Okuhama Y, Shiraishi M, Higa T, Tomori H, Taira K, Mamadi T (1999). Protective effects of ulinastatin against ischemia-reperfusion injury. J Surg Res.

[14] Karnad DR, Bhadade R, Verma PK, Moulick ND, Daga MK, Chafekar ND (2014). Intravenous administration of ulinastatin (human urinary trypsin inhibitor) in severe sepsis: a multicenter randomized controlled study. Intensive Care Med.

[15] Tsujino T, Komatsu Y, Isayama H, Hirano K, Sasahira N, Yamamoto N (2005). Ulinastatin for pancreatitis after endoscopic retrograde cholangiopancreatography: a randomized, controlled trial. Clin Gastroenterol Hepatol.

[16] Wan X, Xie X, Gendoo Y, Chen X, Ji X, Cao C (2016). Ulinastatin administration is associated with a lower incidence of acute kidney injury after cardiac surgery: a propensity score matched study. Crit Care.

[17] Lee B, Lee SY, Kim NY, Rha KH, Choi YD, Park S (2017). Effect of ulinastatin on postoperative renal function in patients undergoing robot-assisted laparoscopic partial nephrectomy: a randomized trial. Surg Endosc.

[18] Li X, Li X, Chi X, Luo G, Yuan D, Sun G (2015). Ulinastatin ameliorates acute kidney injury following liver transplantation in rats and humans. Exp Ther Med.

[19] Khwaja A (2012). KDIGO clinical practice guidelines for acute kidney injury. Nephron Clin Pract.

[20] Kumar AB, Suneja M, Bayman EO, Weide GD, Tarasi M (2012). Association between postoperative acute kidney injury and duration of cardiopulmonary bypass: a meta-analysis. J Cardiothorac Vasc Anesth.

[21] Lagny MG, Jouret F, Koch JN, Blaffart F, Donneau AF, Albert A (2015). Incidence and outcomes of acute kidney injury after cardiac surgery using either criteria of the RIFLE classification. BMC Nephrol.

[22] Cremer J, Martin M, Redl H, Bahrami S, Abraham C, Graeter T (1996). Systemic inflammatory response syndrome after cardiac operations. Ann Thorac Surg.

[23] Farouk A, Hamed RA, Elsawy S, Abd El Hafez NF, Moftah FM, Nassar MAY (2022). Measuring the systemic inflammatory response to on- and off-pump coronary artery bypass graft (CABG) surgeries using the tryptophan/kynurenine pathway. J Invest Surg.

[24] Dey S, Kashav R, Kohli JK, Magoon R, ItiShri W, A.,  (2021). Systemic immune-inflammation index predicts poor outcome after elective off-pump CABG: a retrospective, single-center study. J Cardiothorac Vasc Anesth.

[25] Chen CC, Liu ZM, Wang HH, He W, Wang Y, Wu WD (2004). Effects of ulinastatin on renal ischemia-reperfusion injury in rats. Acta Pharmacol Sin.

[26] Atal SS, Atal S (2016). Ulinastatin–a newer potential therapeutic option for multiple organ dysfunction syndrome. J Basic Clin Physiol Pharmacol.

[27] Lv H, Wei X, Yi X, Liu J, Lu P, Zhou M (2020). High-dose ulinastatin to prevent late-onset acute renal failure after orthotopic liver transplantation. Ren Fail.

[28] Leng Y-X, Yang S-G, Song Y-H, Zhu X, Yao G-Q (2014). Ulinastatin for acute lung injury and acute respiratory distress syndrome: a systematic review and meta-analysis. World J Crit Care Med.

[29] Zhenyu H, Qiaoli Y, Guangxiang C, Maohua W (2022). The effect of Ulinastatin on postoperative course in cardiopulmonary bypass patients in Asia: a meta-analysis of randomized controlled trials. J Cardiothorac Surg.

[30] Oh SY, Kim JC, Choi YS, Lee WK, Lee YK, Kwak YL (2012). Effects of ulinastatin treatment on myocardial and renal injury in patients undergoing aortic valve replacement with cardiopulmonary bypass. Korean J Anesthesiol.

[31] Song J, Park J, Kim JY, Kim JD, Kang WS, Muhammad HB (2013). Effect of ulinastatin on perioperative organ function and systemic inflammatory reaction during cardiac surgery: a randomized double-blinded study. Korean J Anesthesiol.

[32] Song JE, Kang WS, Kim DK, Yoon TG, Kim TY, Bang YS (2011). The effect of ulinastatin on postoperative blood loss in patients undergoing open heart surgery with cardiopulmonary bypass. J Int Med Res.

[33] Chen Q, Hu C, Liu Y, Liu Y, Wang W, Zheng H (2017). Safety and tolerability of high-dose ulinastatin after 2-hour intravenous infusion in adult healthy Chinese volunteers: a randomized, double-blind, placebo-controlled, ascending-dose study. PLoS ONE.

